# Pandemic inequalities: emerging infectious diseases and health equity

**DOI:** 10.1186/s12939-021-01611-2

**Published:** 2022-01-14

**Authors:** Clare Bambra

**Affiliations:** Population Health Sciences Institute, Faculty of Medical Sciences, Ridley Building, Newcastle University, Newcastle upon Tyne, NE1 4LP UK

**Keywords:** Coronavirus, Social determinants of health, Disparities, Socio-economic, Zika, Ebola, COVID-19

## Abstract

The frequency and scale of Emerging Infectious Diseases (EIDs) with pandemic potential has been increasing over the last two decades and, as COVID-19 has shown, such zoonotic spill-over events are an increasing threat to public health globally. There has been considerable research into EIDs – especially in the case of COVID-19. However, most of this has focused on disease emergence, symptom identification, chains of transmission, case prevalence and mortality as well as prevention and treatment. Much less attention has been paid to health equity concerns and the relationship between socio-economic inequalities and the spread, scale and resolution of EID pandemics. This commentary article therefore explores socio-economic inequalities in the nature of EID pandemics. Drawing on three diverse case studies (Zika, Ebola, COVID-19), it hypothesises the four main pathways linking inequality and infectious disease (unequal exposure, unequal transmission, unequal susceptibility, unequal treatment) – setting out a new model for understanding EIDs and health inequalities. It concludes by considering the research directions and policy actions needed to reduce inequalities in future EID outbreaks.

## Introduction

The frequency and scale of Emerging Infectious Diseases (EIDs) with pandemic potential has been increasing over the last two decades and, as COVID-19 has shown, such zoonotic spill-over events are an increasing threat to public health globally. Since 2007, the WHO has made six Public Health Emergency of International Concern (PHIEC) declarations: the 2009 H1N1 influenza pandemic, Ebola (West Africa 2013–2015, Democratic Republic of Congo 2018–2020), poliomyelitis (2014 to present), Zika (2016) and COVID-19 (2020 to present) [1]. There has been considerable research into these PHIECs – especially in the case of COVID-19. However, most of this has focused on disease emergence, symptom identification, chains of transmission, case prevalence and mortality as well as prevention and treatment. Much less attention has been paid to health equity concerns and the relationship between existing socio-economic inequalities and the spread, scale and resolution of EID pandemics [2]. As such, the ‘syndemic’ relationship between inequality and EIDs (how existing socio-economic inequalities interact with- and exacerbate- case rates, symptom severity, morbidity and mortality) remains significantly under explored [3]. This is a surprising oversight given our high understanding of inequalities in other communicable diseases (such as influenza, TB and HIV) as well as the extensive global literature on the social determinants of health in relation to chronic disease [4]. This Commentary article therefore explores the health equity dimensions of EID pandemics. Drawing on three diverse case studies (Zika, Ebola, COVID-19), it hypothesises the pathways linking inequality and infectious disease (unequal exposure, unequal transmission, unequal susceptibility, unequal treatment) – setting out a new model for understanding EIDs and health inequalities. It concludes by considering the research directions and policy actions needed to reduce inequalities in future EID outbreaks.

### EIDs and inequality

The three cases studies Ebola (2015–16, 2018–20), Zika (2016) and COVID-19 (2020 to present) have had diverse reservoir hosts and vectors (mosquitos, bats), various primary modes of transmission (blood, contact, airborne) and impacted on a different range of regions/countries (West Africa, Americas, global). However, they have all resulted in significant social inequalities in terms of morbidity and mortality.

Ebola Virus Disease (EVD), a filoviridae virus, was first identified in 1976 in Zaire (Democratic Republic of Congo). In the 2015–16 outbreak in Guinea, Liberia and Sierra Leone, there were over 28,000 suspected cases and 11,000 deaths. Fruit bats (*Family Pteropodidae*) are considered to be the primary reservoir hosts. Community spread is via blood, bodily fluids and contact. Research has found that transmission was 50% higher in the most impoverished communities and that most of the spread originated in lower socio-economic status areas [4]. In 2014, WHO’s director stated that *“poverty is the mother of the current Ebola epidemic”* [5].

The Zika virus is primarily transmitted by bites from infected mosquitos (*Aedes aegypti* which also carries dengue, chikungunya and yellow fever) as well as from mother to fetus, sexual contact and blood transfusions. It is associated with microcephaly (Congenital Zika Syndrome [CZS]) and Guillain-Barré Syndrome. It was identified in 1947 and the first major outbreak was in French Polynesia in 2013. In 2015–16 it resulted in a pandemic in Brazil and the Americas in which there were over 200,000 suspected cases. Research into microcephaly in Brazil has found strong associations with living conditions: populations with the worst living conditions, had a prevalence ratio for microcephaly more than 5 times higher than those living in areas with the best living conditions [6].

In December 2019, the first cases of an unusual ‘pneumonia’ were documented in the Chinese city of Wuhan. The novel disease, which seems to have jumped from an animal population (horseshoe bats - *Rhinolophus affinis*) into humans, was later named ‘SARS-CoV-2’ or ‘COVID-19’ (coronavirus disease 2019). By January 2020, the disease had started to spread globally, and at the end of the month the World Health Organisation (WHO) declared COVID-19 a Public Health Emergency of International Concern. To date (August 2021), globally, there have been around 200 million recorded cases and over 4 million recorded deaths from COVID-19. Community transmission is primarily by airborne viral droplets. Numerous studies conducted in multiple countries have found strong associations between COVID-19 mortality and socio-economic status with death rates >2  times higher in the most deprived groups [7].

### Pathways to inequality

**Fig. 1:**
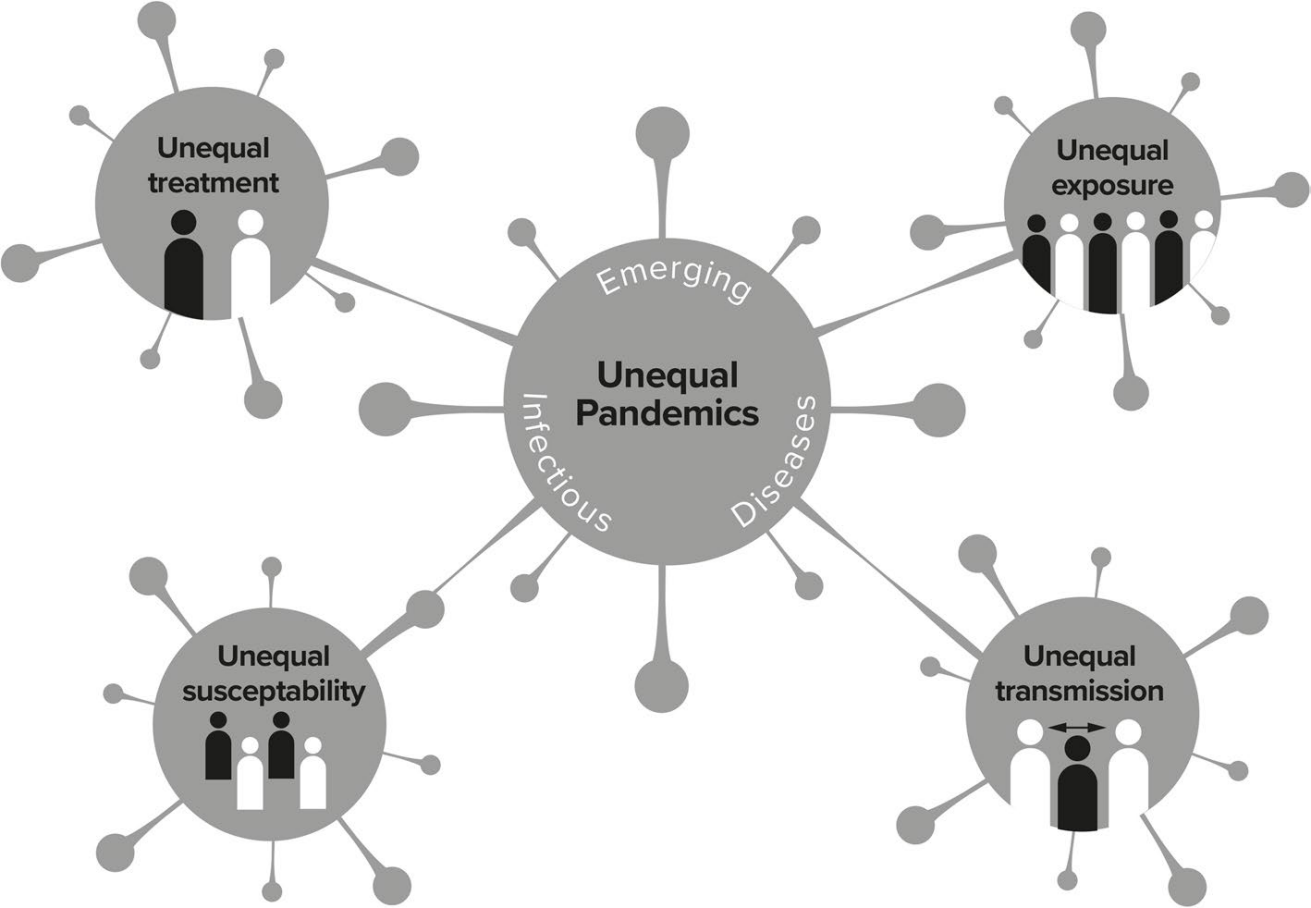
Pathways to Inequalities in Emerging Infectious Diseases Pandemics

Whilst the social epidemiology suggests strong inequalities in EIDs, more thought is needed in terms of understanding how this occurs. Drawing on the three case studies (Zika, Ebola, COVID-19) - and informed by the wider health inequalities, EID and social determinants of health literature [3, 8, 9], this section sets out the four main interconnected pathways (unequal exposure, unequal transmission, unequal susceptibility, unequal treatment) whereby existing social inequalities shape the spread, scale and resolution of EID pandemics (summarised in Fig. 1).

### Pathway 1: unequal exposure

As a result of inequalities in living and working conditions, people from lower socio-economic backgrounds are more likely to be exposed to EID infection (unequal external proximity or contact with a source of a disease agent). For Zika, low income populations living in the Recife favelas were much more likely to be exposed to the disease vector, reporting more mosquito biting relative to residents in other neighbourhoods – mainly due to poor sanitation with more than 90% of favela residents reliant on standing water – a prime location for mosquito breeding [10]. In terms of Ebola, reliance on bush meat consumption amongst the poorest communities may have increased exposure – as has increased encroachment into forests [11]. In terms of COVID-19, lower paid workers were much more likely to be exposed (e.g. by having to continue going into work even during lock downs) [3].

### Pathway 2: unequal transmission

Community transmission (inequality in the passing of a pathogen between community members) is also impacted by the social determinants of health. With Zika, the extreme overcrowding in the urban favelas led to much higher rates of infection [6]. Transmission of COVID-19 was higher in deprived neighbourhoods which had more houses of multiple occupation, smaller house sizes, more urbanity and higher population densities [3]. Differences in health and cultural behaviours also contribute to unequal transmission. For example, in the West African Ebola outbreaks, continuation of traditional funeral practices (including washing and cleaning of the corpse, washing of hands in a common bowl and then touching the face of the deceased) [11] and higher rates of social contacts were noted amongst low income populations [4]. Similarly with Zika, residents of low income neighbourhoods had less knowledge of preventive behaviour measures, perhaps compounded by lower health literacy [10].

### Pathway 3: unequal susceptibility

Unequal susceptibility has two aspects to it. Firstly, pre-existing health conditions (e.g. diabetes, heart disease, obesity, TB, HIV) increase vulnerability to EIDs and can result in more severe symptoms and higher mortality rates post-infection. These co-morbidities are unequally distributed with higher prevalence in more socio-economically deprived populations [8]. Secondly, the social determinants of health also work to make people from low-income communities more vulnerable to EID infection – even when they have no underlying health conditions: living in adverse material (e.g. poor nutrition) and psychosocial circumstances (resulting in chronic stress responses) can exacerbate the onset, course and outcome of infectious diseases. With COVID-19, the higher rates of chronic disease in low socio-economic status communities increased disease severity and mortality rates [3]. For Zika and Ebola, low-income groups had higher pre-existing conditions as well as lower access to health enhancing living conditions (such as good nutrition, sanitation, or housing) [4, 6].

### Pathway 4: unequal treatment

A fundamental factor in inequalities in EIDs is access to health care treatment and preventative services. This was most stark for Ebola where West Africa’s *“intentionally underdeveloped health care infrastructure, legacy of colonial rule, [and] chronic health failures”* [12], resulted in poor access to health care facilities [11]. This was exacerbated by lack of trust in healthcare professionals and resistance to public health approaches (leading to low compliance and reluctance to seek medical care) [11]; reliance on traditional medicine in the management of disease [11]; and the stigmatisation and fear of health care professionals [11]. For Zika, inadequate public health facilities (including disease surveillance) and poor support for CZS cases has been noted [6, 10]. For COVID-19, even in high-income countries with universal health care systems, there is emerging evidence of unequal access to- and uptake of- vaccines [13]. Inequality in access to personal protective equipment (PPE) and inequality in disease testing were also evident in the COVID-19 pandemic [14].

## Conclusion

Whilst not exhaustive, this brief essay has raised concerns that the main health equity aspects of EIDs are overlooked by policymakers and practitioners and under-analysed by researchers [9]. It has outlined a model to understand inequalities in EIDs and it is hoped that this will inspire more careful consideration of equity in EIDs in future research and policy responses. Whilst specific policies will be needed in different countries, contexts and for different EIDs, it is clear that an understanding of the key health equity aspects of EIDs needs to be taken into account in public health responses (e.g. co-designing with local communities and adapting them to local cultural contexts to increase adherence); treatment development (e.g. increasing diversity in clinical trials) and allocation (e.g. training community health workers, ensuring universal accessibility and uptake, proactively identifying areas that need healthcare resources); and prevention (e.g. surveillance systems should include socio-demographic data, ensuring access to vaccines e.g. COVAX). Understanding the social nature of EID pandemics will ultimately help reduce the burden of disease.

## Data Availability

N/A
